# Tuning interfacial fluidity and colloidal stability of membranized coacervate protocells

**DOI:** 10.1038/s42004-024-01193-4

**Published:** 2024-06-03

**Authors:** Yanglimin Ji, Yan Qiao

**Affiliations:** 1 Beijing National Laboratory for Molecular Sciences (BNLMS), Laboratory of Polymer Physics and Chemistry, CAS Research/Education Center for Excellence in Molecular Sciences, Institute of Chemistry, Chinese Academy of Sciences, 100190 Beijing, China; 2 University of Chinese Academy of Sciences, 100049 Beijing, China

**Keywords:** Self-assembly, Origin of life, Colloids

## Abstract

The cell membrane not only serves as the boundary between the cell’s interior and the external environment but also plays a crucial role in regulating fundamental cellular behaviours. Interfacial membranization of membraneless coacervates, formed through liquid-liquid phase separation (LLPS), represents a reliable approach to constructing hierarchical cell-like entities known as protocells. In this study, we demonstrate the capability to modulate the interfacial membrane fluidity and thickness of dextran-bound coacervate protocells by adjusting the molecular weight of dextran or utilizing dextranase-catalyzed hydrolysis. This modulation allows for rational control over colloidal stability, interfacial molecular transport and cell-protocell interactions. Our work opens a new avenue for surface engineering of coacervate protocells, enabling the establishment of cell-mimicking structures and behaviours.

## Abstract (web summary)

Membranization of coacervate microdroplets can stabilize coacervates and enable the construction of hierarchical protocells by using various interfacial layers. Here, the authors demonstrate the modulation of interfacial membrane fluidity and thickness of dextran-bound coacervate protocells by adjusting the molecular weight of dextran or through enzymatic hydrolysis, achieving control over colloidal stability, interfacial molecular transport and cell-protocell interactions.

## Introduction

Coacervation via liquid-liquid phase separation (LLPS) has emerged as a widespread method for constructing membraneless protocell compartments^1–4^. These microcompartments with densely packed interiors, exhibit cell-like behaviors, supporting a range of cytomimetic functions including metabolic reactions^5,6^, genetic transcription and translation^7–9^, growth and division^10–13^, migration^14^, as well as intercellular communication^15–18^. In biology, intracellular membraneless organelles formed through the mechanism of LLPS by various endogenous biomolecules have recently been demonstrated to play an important role in metabolic processes, such as DNA damage repair^19^, transcription regulation^20^, innate immunity^21^, and neurotransmitter release^22^. The study of liquid-like coacervates has therefore attracted significant attention due to their potential functions, including enriching guest clients^23,24^, generating chemical gradients^25^, hosting biochemical reaction networks^26,27^, and delivering therapeutic cargos^28^. However, the lack of a protective layer on coacervates results in weak colloidal stability and limited control over molecular transport across the interface. To tackle this challenge, a range of interfacial layers consisting of surfactants^29,30^, lipids^31–34^, amphiphilic polymers^35,36^, proteins^37,38^, polysaccharides^39^, nanoparticles^40^, fragments of natural cell membranes or cell walls^41,42^ have been employed for coacervate membranization. These meticulously designed molecules or natural extracts have demonstrated the ability to stabilize coacervate protocells against coalescence and provide adaptive interfacial semi-permeability^43^.

In this work, we investigate the interfacial fluidity and membrane thickness of the polysaccharide layer on the surface of coacervates, highlighting their influence on the colloidal stability of coacervate microdroplets. In particular, we showcase the modulation of interfacial molecular transport and resistance to bacterial invasion through variations in dextran molecular weights or enzymatic hydrolysis, breaking dextran into smaller fragments. Our findings present a membranization strategy for tailoring the surface properties of coacervate microdroplets and highlight the interfacial properties governing the colloidal stability, which allows for the construction of hierarchical coacervate protocells and prototissues.

## Results and discussion

### Colloidal stability of dextran-coated coacervate protocells

**Fig. 1:**
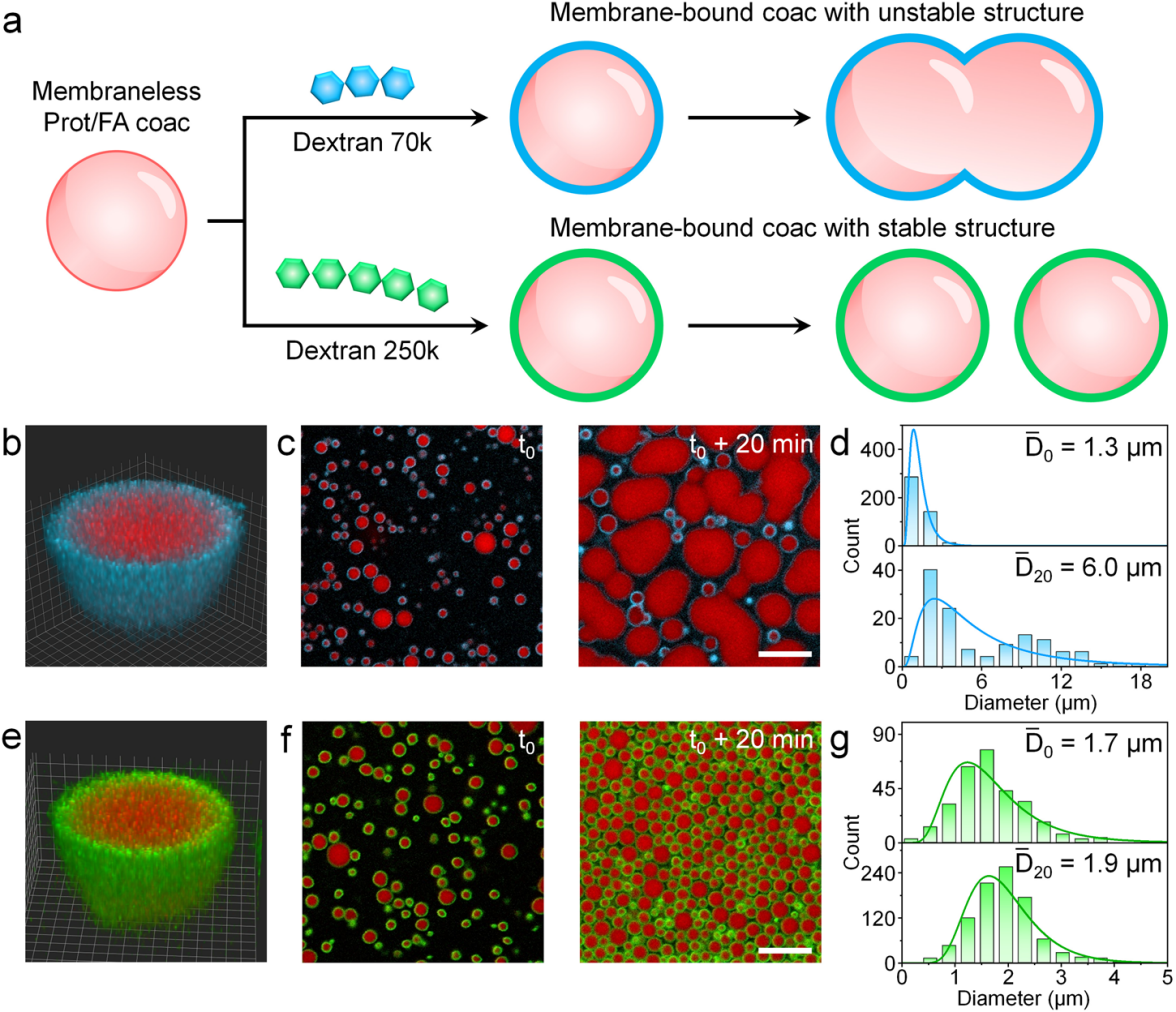
Structural stability of dextran-bound coacervate protocells. **a**–**g** Structural changes in coacervate microdroplets upon coating with dextran 70k (**b**–**d**) and dextran 250k (**e–g**): **a** schematic illustration, **b**, **e** reconstructed 3D CLSM images, **c**, **f** time-lapse fluorescence microscopy images, and **d**, **g** size distributions of dextran-membranized Prot/FA coacervates. The freshly prepared dextran 70k-bound coacervates underwent coalescence over 20 min and showed an increase in mean diameters from 1.3 to 6.0 μm, while dextran 250k-bound coacervates remained largely unchanged in droplet size. Prot/FA coacervates were doped with rhodamine B (0.5 μM, red) and dextran was labeled with FITC (blue in **b**, **c** and green in **e**, **f**). The mean diameters were determined by the statistic of coacervates in (**c**, **f**). *D*_0_ and *D*_20_ represented the mean diameters at *t*_0_ and *t*_0_ + 20 min, respectively. Grid width, 0.5 μm. Scale bars, 10 μm.

To enhance the colloidal stability, we initially explored the membranization of liquid-like coacervate protocells formed by the phase-separated protamine/folic acid mixture (Prot/FA, molar ratio of 5:1) at a total concentration of 25 mM. We achieved this by direct interfacial assembly of dextran molecules on the surface (Fig. 1a). For visualization purposes, fluorescein isothiocyanate (FITC) labeled dextran 70 kDa (0.32 mg/ml) was assembled on the Prot/FA coacervates. Confocal laser scanning microscopy (CLSM) image showed the formation of a continuous dextran membrane (green fluorescence, Fig. 1b). Despite uniform coating with dextran, these polysaccharide-bound coacervates displayed colloidal instability, which fused with neighboring microdroplets and showed significant size increase within 20 min, with mean diameter growing from 1.3 to 6.0 μm (Fig. 1c, d and Supplementary Movie 1). Notably, increasing the molecular weight of dextran (Mw 250 kDa, 0.32 mg/ml) resulted in the formation of a stable membrane on Prot/FA coacervates, preventing the fusion of coacervate microdroplets for more than 72 h (Fig. 1e, f, Supplementary Fig. 1 and Supplementary Movie 2). After incubation for 20 min, the complex coacervates coated with dextran 250k remained isolated, and the size of microdroplets were largely unchanged (~ 1.9 μm, Fig. 1g). Consistent results were obtained with varied molecular weights of dextrans, in which dextran 40k failed to protect coacervates from fusion, whereas dextran 500k was found to strongly stabilize coacervates (Supplementary Fig. 2).

**Fig. 2:**
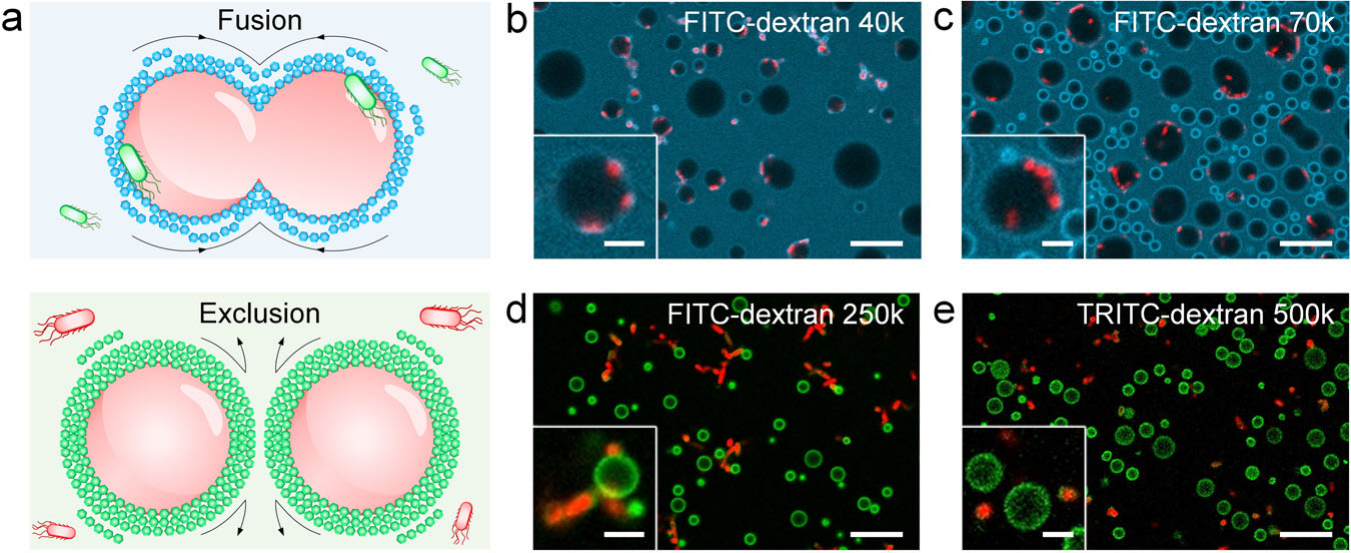
Membranized coacervate microdroplets against bacteria invasion. **a** Schematic illustration of distinct bacteria-coacervate interactions regulated by dextran with varied molecular weights. The Prot/FA coacervate microdroplets protected by low molecular weight dextran underwent droplet fusion and *E. coli* sequestration, while surface coating with high molecular weight dextran enhanced the structural stability of coacervates and excluded the internalization of *E. coli*. **b**–**e** CLSM images showing *E. coli* cells interacting with dextran-coated coacervates with dextran 40k (**b**), dextran 70k (**c**), dextran 250k (**d**) and dextran 500k (**e**). The *E. coli* cells expressed RFP (**b**–**d**) or EGFP (**e**) proteins. Scale bars, 5 μm and 1 μm (insets).

We then investigated the interaction between dextran-bound coacervates and various guest species including small molecule, proteins, and bacteria (Fig. 2a). Specially, we examined the permeability of these guests into dextran-coated Prot/FA coacervates, evaluating the effect of the molecular weight of polysaccharide. Hoechst 33258 and bovine serum albumin (BSA) were found to permeate the dextran membranes, being sequestered by the coacervate lumens (Supplementary Fig. 3). In addition, we expressed red fluorescence protein (RFP) or enhanced green fluorescence protein (EGFP) in *Escherichia coli* (*E. coli*) cells and directly visualized the cell-protocell interactions under microscope. We noticed that *E. coli* were able to invade the complex coacervates protected by dextran 40k and 70k (Fig. 2b, c), while coacervate microdroplets protected by dextran 250k and dextran 500k prevented the bacteria invasion (Fig. 2d, e).

### Dextranase-regulated colloidal stability of dextran-membranized coacervates

**Fig. 3:**
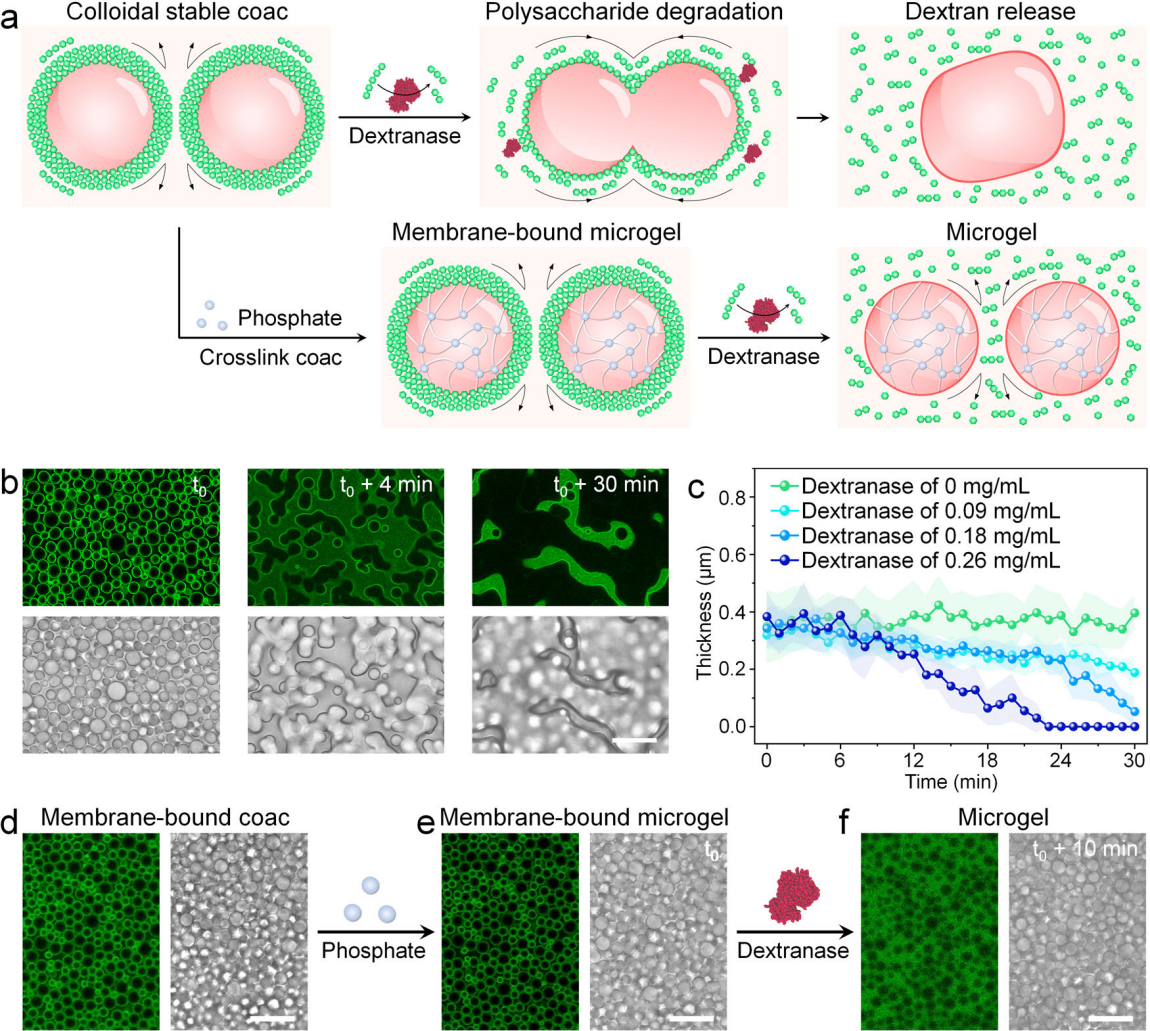
Dextranase-induced coalescence of membrane-bound coacervates and the defence strategy. **a** Upper row: schematic illustration of the dextranase-triggered degradation of dextran membrane on coacervates, resulting in the generation of small dextran fragments distributed in the surroundings and the coalescence of coacervate microdroplets. Lower row: scheme showing the transformation of membrane-bound coacervate to membrane-bound microgel via crosslinking by phosphate, which resisted the coalescence of protocells after membrane digestion by dextranase. **b** Time-series fluorescence (upper row) and optical microscopy images (lower row) depicting the gradual degradation of FITC-dextran 250k membrane (green fluorescent region) and fusion of Prot/FA coacervates (non-fluorescent region) after the addition of dextranase (0.18 mg/ml), accompanied by the liberation of dextran fragments into the surrounding solution. Scale bar, 5 μm. **c** Time-dependent variation of membrane thickness upon the addition of dextranase with final concentrations of 0, 0.09. 0.18, and 0.26 mg/ml, leaving residual thicknesses of 0.39, 0.17, 0.03, and 0 μm after 30 min, respectively. The average diameter of coacervate microdroplets was determined by measuring 20 microdroplets and the error curves represented the standard deviation. **d**–**f** Fluorescence (left) and optical microscopy images (right) for FITC-dextran 250k membranized coacervates (**d**), membrane-bound microgel (**e**) and membraneless microgel (**f**). The transformation process was realized by sequential addition phosphate and dextranase. The microgel did not coalesce after the degradation of membrane. Scale bars, 10 μm.

Furthermore, the colloidal stability of polysaccharide-protected coacervates was lowered in the presence of dextranase, which enzymatically degraded dextran into small fragments. This enzymatic hydrolysis resulted in a reduction in the thickness of the polysaccharide membrane, weakening the colloidal stability of the membrane-bound coacervates and leading to their coalescence (Fig. 3a). Specifically, the membrane thickness of the dextran (Mw = 250k) stabilized Prot/FA coacervates decreased upon incubation with 0.18 mg/ml dextranase, releasing the interfacial FITC-dextran to the surroundings, and causing the slow fusion of coacervate microdroplets (Fig. 3b and Supplementary Movie 3). Significantly, the effect of membrane degradation was influenced by the concentration of dextranase. The thickness of dextran membrane decreased faster with the increase of dextranase concentration of 0.09 to 0.18, and 0.26 mg/ml, leading to a relative decrease of residual thicknesses to 0.17, 0.03 and 0 μm after 30 min, respectively (Fig. 3b, c and Supplementary Fig. 4a, b). In contrast, the membrane thickness remained unchanged in the absence of dextranase (Fig. 3c and Supplementary Fig. 4c).

We also altered the molecular weights of dextran to investigate dextranase-triggered coalescence. Dextrans with lower molecular weights (10k to 70k) showed a reduced tendency to adsorb on the surface of coacervates and were less effective in stabilizing coacervate microdroplets (Supplementary Fig. 5). Dextrans with higher molecular weights (500k) increased the stability of membranized coacervates, which retained a relative thick residual membrane (0.25 μm) after enzymatic hydrolysis for 30 min (Supplementary Figs. 4d and 6).

We further developed a strategy for membrane-bound Prot/FA coacervate protocell to resist the coalescence after dextranase-mediated hydrolysis of membrane (Fig. 3a). To this end, phosphates (1 mM, pH 7.0) were utilized to crosslink the Prot/FA coacervates into microgels via multivalent electrostatic interactions. The addition of phosphates did not change the spherical shapes of protocells (Fig. 3d, e) but stabilized the microgels in the presence of dextranase (Fig. 3f and Supplementary Movie 4).

### Molecular weight related membrane fluidity and thickness

**Fig. 4:**
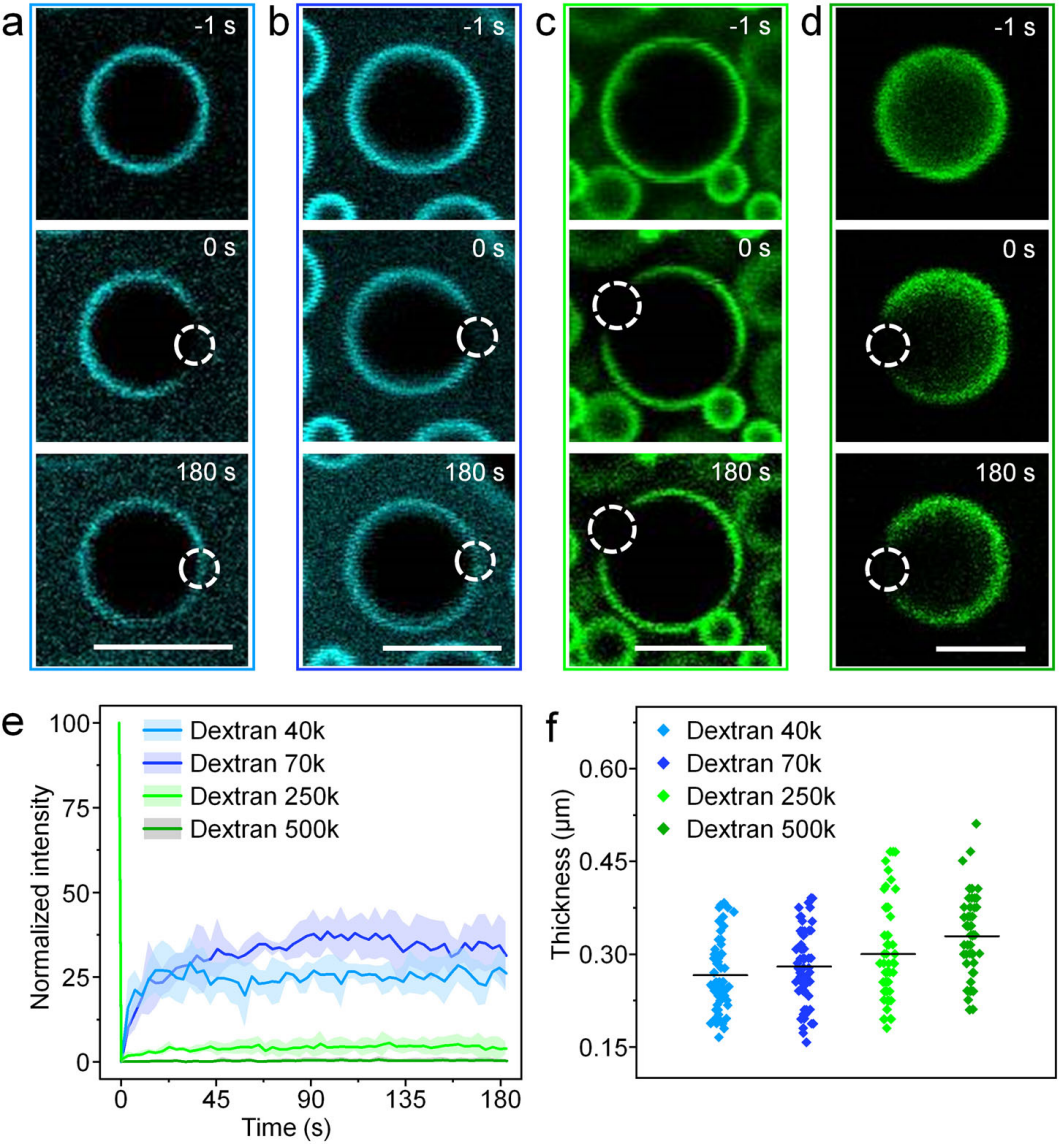
Molecular weight-dependent membrane property on coacervates. **a**–**e** Time-lapse fluorescence microscopy images (**a**–**d**) and fluorescence intensity changes (**e**) after photobleaching on the dextran membranes at surface of Prot/FA coacervates. The molecular weight of dextran was 40k (**a**, FITC, blue), 70k (**b**, FITC, blue), 250k (**c**, FITC, green), and 500k (**d**, TRITC, green). The white circles indicated the bleaching geometries (regions of interest). The recovery of fluorescence intensity after photobleaching was found to be slower when the molecular weight of dextran increased, demonstrating the decrease of the membrane fluidity on Prot/FA coacervates. Error curves represented the standard deviation in three replicating measurements. Scale bars, 5 μm. **f** Thickness statistics of the dextran membranes on coacervates with varied dextran molecular weights of 40, 70, 250, and 500 kDa, showing an increase in thickness of 0.27, 0.28, 0.30 and 0.33 μm, respectively.

To elucidate the structural factors influencing the colloidal stability and interfacial permeability of membrane-bound coacervates, we conducted a thorough investigation into the fluidity and thickness of dextran membranes on the surface of Prot/FA coacervates. Employing fluorescence recovery after photobleaching (FRAP), we examined the molecular diffusion of polysaccharides within the interfacial membrane. Fast fluorescence recoveries were observed after photobleaching, with 25% and 30% recovery within 10 and 50 s, on the membranes of dextran 40k and 70k (Fig. 4a, b, e), giving estimated apparent diffusion coefficients of 1.0 × 10^−14^ and 3.1 × 10^−14^ m^2^ s^–1^, respectively. In contrast, the dextran 250k and 500k membranes on coacervate microdroplets exhibited negligible fluorescence recoveries after photobleaching (Fig. 4c–e). Meanwhile, the statistical membrane thicknesses of dextran 40k, 70k, 250k, and 500k surrounding Prot/FA coacervates were qualitatively measured by using a super-resolution light microscopy, which gave average values of 0.27, 0.28, 0.30, and 0.33 μm, respectively, indicative of a positive correlation between membrane thickness and molecular weight (Fig. 4f). On the basis of these results, we proposed that the high membrane fluidity induced interfacial defects, which facilitated the guest species to permeate across the membrane. The membrane thickness affected the protective effect of the physical barrier, where a thick membrane exhibited a better performance in inhibiting the coacervate coalescence.

## Conclusions

In conclusion, the interfacial assembly of a dextran layer on the surface of Prot/FA coacervates played a crucial role in regulating the colloidal stability of coacervate microdroplets. Notably, increasing the molecular weight of dextran led to reduced fluidity and increased thickness of the dextran membrane. This phenomenon was attributed to the elongation of the polysaccharide chain, which strengthened the multivalency of charge-dipole interaction between positive charge and hydroxyl groups, as well as the hydrogen bonding between dextrans and coacervates^39,44^, resulting in the formation of a thick rigid membrane. Coacervate microdroplets protected by low molecular weight dextran (40k and 70k) failed to stabilize the coacervates or prevent the invasion of *E. coli* cells. In contrast, dextran 250k and 500k robustly protected the coacervates from coalescence and effectively excluded *E. coli* cells. Additionally, the introduction in dextranase was shown to trigger the degradation of dextran, causing a reduction of membrane thickness and a loss in the coacervates stability. This study offered an effective methodology to regulate the interfacial property of complex coacervates and modulate the interactions between synthetic and living cells, paving the way toward the design of cytomimic structures and functions.

## Methods

### Preparation of dextran-coated Prot/FA coacervate microdroplets

Typically, the Prot/FA coacervate microdroplets were prepared by mixing 20 μl of FA (11.08 mg/ml, 25 mM, pH = 10.0) and 100 μl of Prot solution (4.25 mg/ml, average molecular weight of amino acid of 170 Da, estimated monomer concentration of 25 mM, pH = 10.0). For the membranization of Prot/FA coacervates, 4 μl of FITC-dextran 10k, 20k, 40k, 70k, 250k, or TRITC-dextran 500k solution (10 mg/ml) was added into 120 μl of Prot/FA coacervate dispersion followed by gently stirring with a pipette tip to ensure homogeneously coating. All experiments were repeated three times.

### Fluorescence imaging of coacervate microdroplets

Fluorescence microscopy experiments were undertaken by using a confocal laser scanning microscopy (CLSM, Zeiss LSM880, Germany) with a ×63 oil immersion lens. Fluorophores were excited with an Argon laser (488 nm for FITC) and a HeNe543 laser (543 nm for RITC and TRITC). Detection bands were set at 500‒550 nm for FITC and 550‒630 nm for RITC and TRITC.

Super-resolution light microscopy imaging was performed using a Leica Stellaris 8 Confocal Microscope with an integrated Lightning detection using a 63x oil immersion objective (NA = 1.4). Lightning is a Leica ultra-high-resolution confocal platform, which is based on adaptive computational deconvolution and has overcome the resolution down to 120 nm with simultaneous multicolor imaging. Qualitative/relative thickness measurement were performed with it due to the limitations of confocal from the Point Spread Function (PSF) and NA, etc.

### Statistics of coacervate size distribution and membrane thickness

The size distributions and average diameter changes of dextran-bound coacervates were determined by analyzing the time-series super-resolution light microscopy images with ImageJ software. The thicknesses were measured by the distances of half peak heights from line scan data, where the fluorescence intensity of surroundings was set as the baseline. Data were collected from the super-resolution light microscopy images of freshly prepared membrane-bound coacervates using ImageJ software.

### Guest species sequestration by membrane-bound Prot/FA coacervates

One hundred twenty μl of dextran-coated Prot/FA suspensions were mixed with 3 μl of Hoechst (10 μM), RITC-BSA (5 mg/ml) or RFP-E. coli suspension (OD_600_ = 1.8) for the permeability investigation of FITC-dextran 40k, 70k and 250k membrane, and 3 μl of Hoechst (10 μM), FITC-BSA (5 mg/ml), EGFP-E. coli (OD_600_ = 1.8) for the permeability investigation TRITC-dextran 500k membrane. All experiments were repeated three times.

### Dextranase-mediated dextran membrane degradation

Twenty μl of FITC-dextran 250k-coated Prot/FA coacervate suspension was added into a glass slide followed by the addition of 1, 2, or 3 μl of dextranase (2 mg/ml) to initiate the degradation of the membrane. For TRITC-dextran 500k-coated Prot/FA coacervates, 2 μl of dextranase (2 mg/ml) was added to trigger the hydrolysis. The fluorescence images during the degradation and coacervate fusion were acquired by a super-resolution light microscopy (Leica Stellaris 8 Confocal Microscope) every 10 s. All experiments were repeated five times.

### Phosphate-mediated resistance of dextranase-induced coalescence

Typically, 2 μl of phosphate solution (20 mM of sodium dihydrogen phosphate and sodium dihydrogen phosphate mixture, pH 7.0) was added into 20 μl of FITC-dextran 250k-coated Prot/FA coacervate suspension to trigger the gelation. Then 3 μl of dextranase (2 mg/ml) was added to trigger the hydrolysis. The fluorescence and bright field images during the degradation was collected by a confocal microscope (Zeiss LSM880) every 10 s. All experiments were repeated three times.

### Fluorescence recovery after photobleaching (FRAP)

FRAP experiments consisted of a bleaching stage obtained by exposing the region of interest (ROI) to a 100% power laser beam for 1 s, followed by a recovery stage monitored with a 5% power laser beam. Fluorescence images were acquired using a confocal microscope (Olympus FV1000-IX81, iXon camera) with a ×60 oil immersion lens and a 559 nm laser for TRITC, and a 488 nm laser for FITC. Time sequences of fluorescence images were collected every 4 s, starting right after the photobleaching event. The apparent diffusion coefficients (*D*_app_) were calculated according to $\frac{{{{r}}}^{2}}{{{t}}}$, where *t* is the recovery time after photobleaching, and *r* is the radius of ROI. All experiments were repeated three times.

### Reporting summary

Further information on research design is available in the Nature Portfolio Reporting Summary linked to this article.

### Supplementary information

Supplementary Information

Description of Additional Supplementary Files

Supplementary Data

Supplementary Movie 1

Supplementary Movie 2

Supplementary Movie 3

Supplementary Movie 4

Reporting Summary

## Data Availability

All data and needed to evaluate the conclusions in the paper are present in the article and/or the Supplementary Information (including Supplementary Methods and Supplementary Figs.), Supplementary Data and Supplementary Movie files. Additional data related to this study can be obtained from the corresponding authors upon request.
