# Supplementary material for: Tuning interfacial fluidity and colloidal stability of membranized coacervate protocells
Source: Commun Chem. 2024 Jun 3;7:122. doi: 10.1038/s42004-024-01193-4 (PMC11148010; doi:10.1038/s42004-024-01193-4)
Supplement: Supplementary file 1 — Supplementary Information [file 42004_2024_1193_MOESM1_ESM.pdf]

Supplementary information for

Tuning interfacial fluidity and colloidal stability of membranized coacervate protocells

Yanglimin Ji[1 2], Yan Qiao[1 2,*]

[1]Beijing National Laboratory for Molecular Sciences (BNLMS), Laboratory of Polymer Physics and Chemistry, CAS Research/Education Center for Excellence in Molecular Sciences, Institute of Chemistry, Chinese Academy of Sciences, Beijing 100190, China

[2]University of Chinese Academy of Sciences, Beijing 100049, China

[*]Correspondence author: yanqiao@iccas.ac.cn

Supplementary Methods

Materials. Protamine sulfate salt (Prot, from *salmon*, Sigima), folic acid (FA, Ark Pharm), bovine serum albumin (BSA, ≥98%, M_w = 66.4 kDa, Sigma), 1,6-α-D-dextran 6-dextranohydrolase (dextranase, 100-250 units/mg protein, Sigma), isopropyl β-D thiogalactosidesodium (IPTG, Sangon Biotech, ≥99.0%), streptomycin (Sangon Biotech, 98%), sodium phosphate monobasic (NaH_2PO_4, Beijing Shiji, ≥99.5%), and sodium phosphate dibasic anhydrous ($Na_2HPO_4·3H_2O$, Beijing Shiji, ≥99%), were used as received. Other chemicals were purchased from the Beijing Chemical Works. Deionized water (specific resistance of 18.2 MΩ•cm, Milli-Q Reference, Millipore, USA) was used in all the experiments.

Fluorescent molecules. Fluorescein isothiocyanate-dextran (FITC-dextran 10 kDa, 20 kDa, 40kDa, 70 kDa, and 250 kDa, Sigma), tetramethylrhodamine isothiocyanate-dextran (TRITC-dextran 500 kDa, Sigma), rhodamine B (Aladdin), Hoechst 33258 (Hoechst, Invitrogen), fluorescein isothiocyanate (FITC, ≥90% HPLC, Sigma), rhodamine B isothiocyanate (RITC, mixed isomer, Sigma), and 1-Pyrenemethylamine hydrochloride (pyrene, Sigma) were used as received.

Fluorescent labelling of proteins. In brief, 10 mg BSA was dissolved in 5 mL of sodium carbonate buffer (100 mM, pH 8.5). 250 μL of FITC solution (1.0 mg/mL, in DMSO) was then added slowly in 5 μL aliquots. The mixture was stirred at room temperature overnight, followed by purification with dialyzing (Medicell dialysis tubing, MWCO 12–14 kDa) against Milli-Q

water for 1 day and concentrated with Millipore Ultra centrifugal filter (10 kDa). RITC-BSA was labelled with the same procedures.

Culture of E. coli cells. The E. coli BL21(DE3) with pCDF-*EGFP* or pCDF-*RFP* cells were grown in Luria-Bertani medium containing streptomycin (50 μg/mL) at 37 °C. When the OD_{600} of the cell culture reached 0.6-0.8, 100 μM IPTG was added, followed by culturing for 20 h at 16 °C. After 20 h, cells were harvested by centrifuging at 7000 g for 3 min and washed with Milli-Q water 3 times.

Supplementary Figures

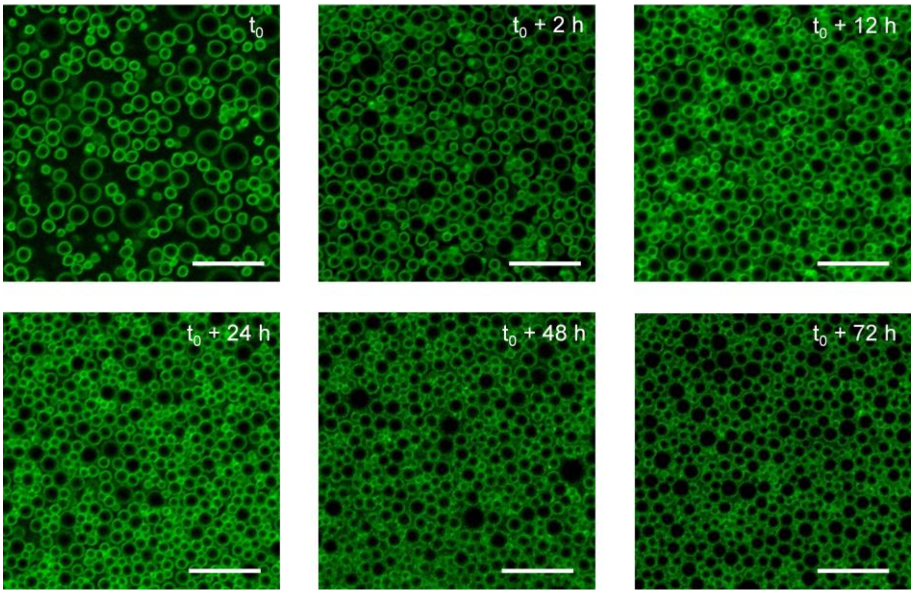

Supplementary Figure 1. Fluorescence microscopy images of FITC-dextran 250k-membranized Prot/FA coacervate microdroplets, which remained stable after 72 h. Scale bars, 10 μm.

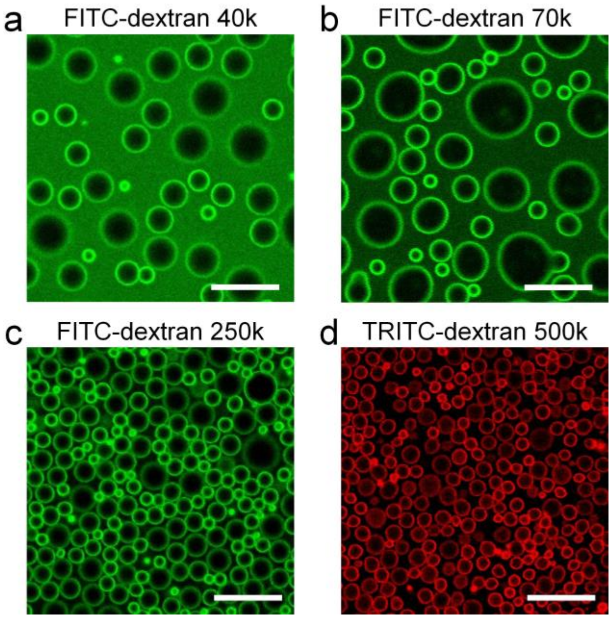

Supplementary Figure 2. (**a-d**) CLSM images of Prot/FA coacervates membranized with FITC-dextran 40k (**a**), 70k (**b**), 250k (**c**), and TRITC-dextran 500k (**d**). Coacervates underwent fusion when membranized with FITC-dextran 40k (**a**) and 70k (**b**), while were stabilized with FITC-dextran 250k (**c**) and TRITC-dextran 500k (**d**). Scale bars, 20 μm.

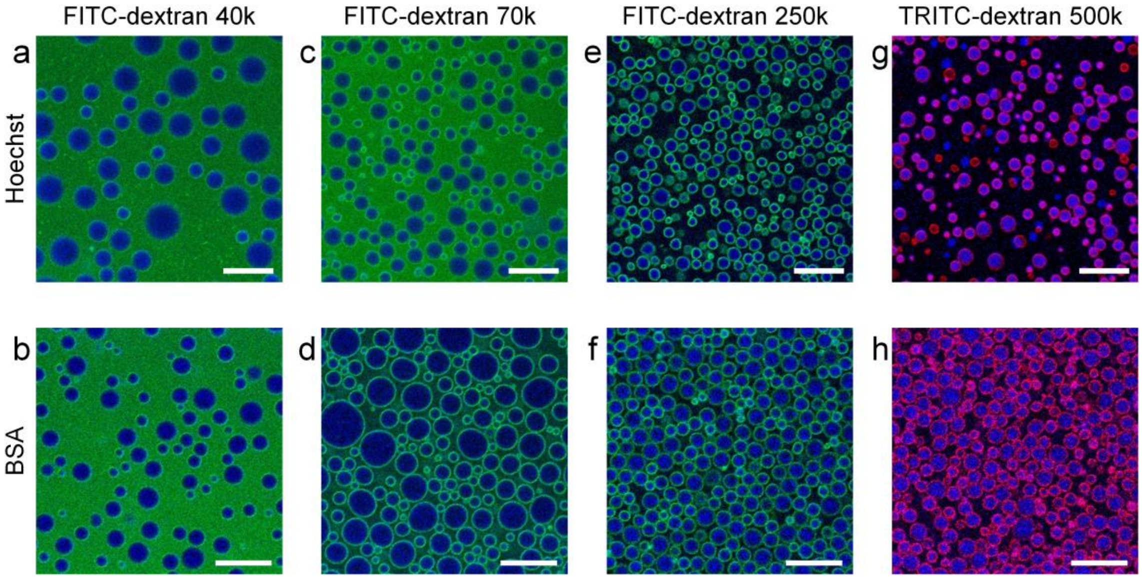

Supplementary Figure 3. (**a-h**) Fluorescence microscopy images for the membranized coacervates by FITC-dextran with different molecular weights of 40k (**a,b**), 70k (**c,d**), 250k (**e,f**), and TRITC-dextran 500k (**g,h**) which were mixed with aqueous solutions of Hoechst (**a,c,e,g**), and pyrene-BSA (**b,d,f,h**). Hoechst and BSA were sequestered by the coacervate lumen. Scale bars, 10 μm.

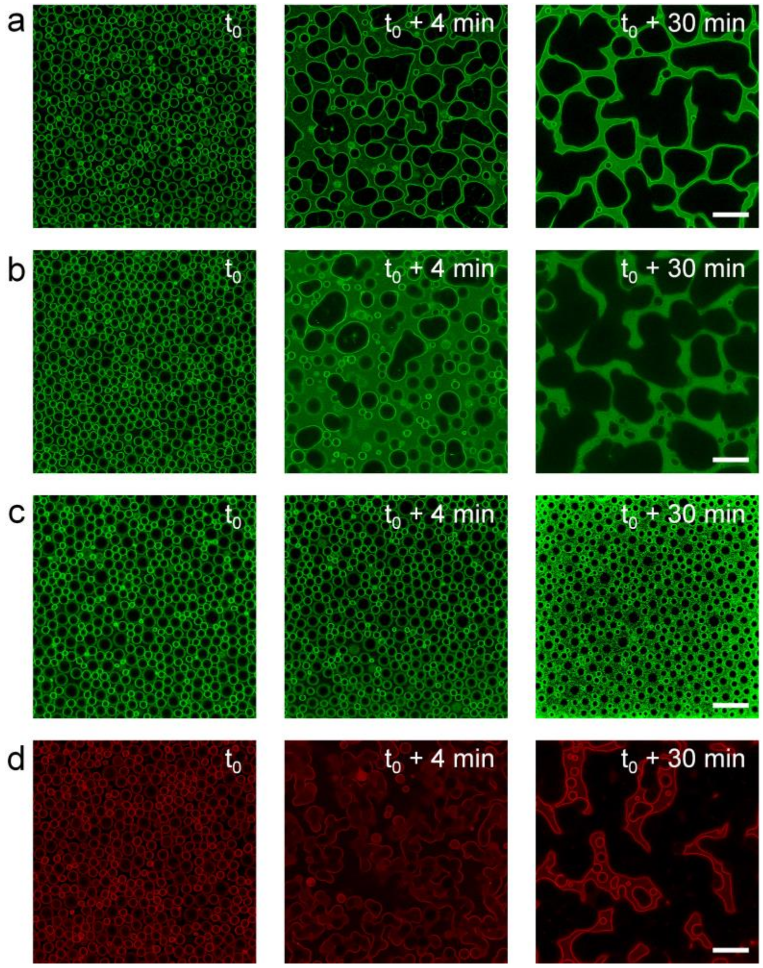

Supplementary Figure 4. (**a**-**c**) Time-series CLSM images of FITC-dextran 250k-bound coacervates upon adding 0.09 mg/mL (**a**), 0.26 mg/mL (**b**), and 0 mg/mL (**c**) of dextranase. (**d**) CLSM images of TRITC-dextran 500k-bound coacervates incubating with 0.18 mg/mL of dextranase (**d**). Scale bars, 5 µm.

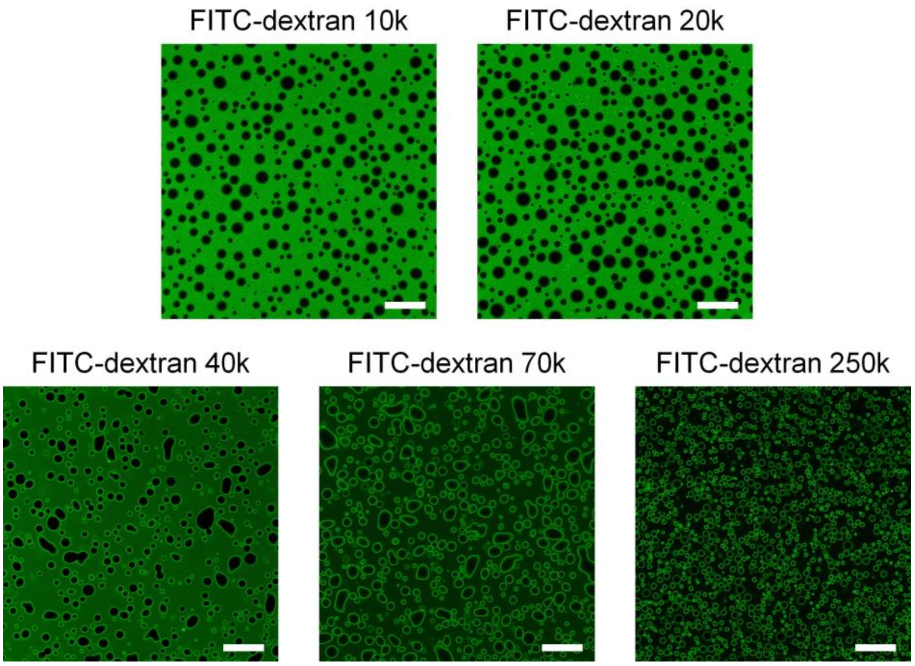

Supplementary Figure 5. Fluorescence microscopy images of Prot/FA coacervate microdroplets membranized with FITC-dextran 10k, 20k, 40k, 70k, and 250k. The aggregation of dextran molecules on the coacervate surface increased with the molecular weight increased, generating a thicker membrane. Scale bars, 20 μm.

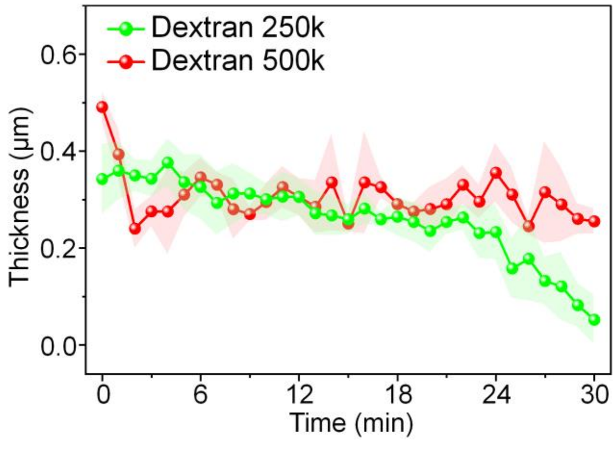

Supplementary Figure 6. Time-dependent changes of membrane thickness upon the addition of dextranase (0.18 mg/mL) into FITC-dextran 250k (green curve) and TRITC-dextran 500k-bound coacervates (red curve), respectively. The residual membrane of dextran 500k was thicker after 30 min digestion.
