# Supplementary material for: Tuning interfacial fluidity and colloidal stability of membranized coacervate protocells
Source: Commun Chem. 2024 Jun 3;7:122. doi: 10.1038/s42004-024-01193-4 (PMC11148010; doi:10.1038/s42004-024-01193-4)
Supplement: Supplementary file 2 — Description of Additional Supplementary Files [file 42004_2024_1193_MOESM2_ESM.pdf]

Description of Additional Supplementary Files

File name: Supplementary Data

File Description: the numerical source data for graphs.

File name: Supplementary Movie 1.

File Description: Fluorescence microscopy video showing the fusion of FITC-dextran 70k-bound Prot/FA coacervates containing rhodamine B. The movie was shown at ×100 of real-time speed at 5 frames per second. The total duration of the recording was 20 min;real time was shown at the top right. Scale bar, 10 µm.

File name: Supplementary Movie 2.

File Description: Fluorescence microscopy video showing the FITC-dextran 250k membrane-stabilized rhodamine Bdoped Prot/FA coacervates resisted fusion. The movie was shown at ×100 of real-time speed at 5 frames per second. The total duration of the recording was 20 min; real time was shown at the top right. Scale bar, 10 µm.

File name: Supplementary Movie 3

File Description: Fluorescence (left) and optical microscopy video (right) showing the dextranase-mediated FITC-dextran 250k membrane degradation and subsequent coacervate fusion. The movie was shown at ×50 of real-time speed at 10 frames per second. The total duration of the recording was 30 min; real time was shown at the top right. Scale bar, 5 µm.

File name: Supplementary Movie 4.

File Description: Fluorescence (left) and optical microscopy (right) video showing the microgel kept isolated after adding dextranase to hydrolyze the membrane. The movie was shown at ×60 of real-time speed at 5 frames per second. The total duration of the recording was 10 min; real time was shown at the top right. Scale bar, 10 µm.
